# Clinical Validation of DNA Extraction-Free qPCR, Visual LAMP, and Fluorescent LAMP Assays for the Rapid Detection of African Swine Fever Virus

**DOI:** 10.3390/life12071067

**Published:** 2022-07-16

**Authors:** Lili Yang, Lin Wang, Meihui Lv, Yu Sun, Jijuan Cao

**Affiliations:** 1 Key Laboratory of Biotechnology and Bioresources Utilization of Ministry of Education, Dalian Minzu University, Dalian 116600, China; lanborui0026@vip.163.com (L.Y.); 18647597633@163.com (M.L.); 2 Beijing Animal Disease Prevention and Control Center, Beijing 102629, China; lwang0631@163.com; 3 China Animal Disease Prevention and Control Center, Beijing 102206, China

**Keywords:** African swine fever virus, qPCR, visual LAMP, fluorescent LAMP, DNA extraction-free, clinical validation, rapid detection

## Abstract

The global pig industry and food safety are seriously threatened by outbreaks of African swine fever (ASF). To permit early diagnosis of African swine fever virus (ASFV), prevent its spread, and limit its outbreaks, a highly sensitive diagnostic method that can be performed at pig farms is required. Herein, we established DNA extraction-free real-time PCR (qPCR), visual loop-mediated isothermal amplification (LAMP), and fluorescent LAMP assays, which were compared with the results of World Organization for Animal Health (OIE) qPCR to assess ASFV-infected clinical samples. Based on plasmid DNA, the limit of detection for the three assays and OIE qPCR were 5.8 copies/μL. All four assays had good ASFV specificity and showed no cross-reactivity with other tested viruses. These assays were used to diagnose 100 clinical samples. The assays showed good diagnostic consistency, with kappa values of 1.0, 0.84, and 0.88, respectively. Compared with OIE qPCR, the diagnostic specificity/sensitivity of DNA extraction-free qPCR, visual LAMP, and fluorescent LAMP assays were 100%/100%, 100%/87.1%, and 100%/90.32%, respectively. The assays eliminated the need for DNA extraction and are more suitable for ASF diagnosis by inexperienced farmers in low-resource environments, making them a good choice for on-site monitoring of pig farms.

## 1. Introduction

African swine fever (ASF) is an infectious disease of domestic and wild pigs of all breeds and ages, caused by African swine fever virus (ASFV). The clinical syndromes vary from per-acute, acute, and subacute to chronic, depending on the virulence of the virus [1]. Acute disease is characterized by high fever, hemorrhages in the reticuloendothelial system, and a high mortality rate [2,3].

The current distribution of ASF extends across more than 50 countries in three continents (Africa, Asia, and Europe). Several incursions of ASF out of Africa were reported between the 1960s and 1970s. In 2007, ASF was introduced into Georgia, from where it spread to neighboring countries including the Russian Federation. From there, ASF spread to Eastern European countries, extending westwards and reaching the European Union in 2014. Further westward and southern spread in Europe has occurred since that time. In all these countries, both hosts—domestic pig and wild boar—were affected by the disease. In August 2018, China reported an ASF epidemic for the first time [4,5], which was followed by infection in other Asian countries [6]. ASF has a high risk of damaging the global pig industry and adversely affecting food safety [7].

ASFV is a double-stranded DNA virus, with a large and complex genome (170–193 kb) that encodes 160–170 genes [8,9]. It is currently classified as the only member of the Asfaviridae family, genus Asfivirus [10]. More than 60 structural proteins have been identified in intracellular virus particles (200 nm) [11]. The complete genomes of several ASFV strains have been sequenced [12,13,14]. Based on the EP402R gene (encoding serotype specific protein CD2v) and ASFV B646L gene (encoding capsid protein p72), 8 serogroups and 24 different genotypes were identified, respectively [15,16]. Different strains of ASFV vary in their ability to cause disease, but at present, there is only one recognized serotype of the virus detectable by antibody tests. ASF epidemiology is complex, with different epidemiological patterns of infection occurring in Africa and Europe. ASF occurs through transmission cycles involving domestic pigs, wild boar, wild African suids, and soft ticks [17]. ASF is a notifiable disease to the World Organization for Animal Health (OIE). At present, Vietnamese scientists have made major breakthroughs in the research and development of new vaccines, which has played a great role in promoting the development of ASF vaccine. At present, Vietnamese scientists have made major breakthroughs in the research and development of new vaccines, which has played a great role in promoting the development of African swine fever vaccine. However, early diagnosis and isolation are still the conventional methods to control ASFV transmission [18,19].

Fluorescent quantitative real-time PCR (qPCR) assay has been widely used to detect ASFV sensitively and specifically [20]. However, because of the complexity of the nucleic acid isolation methods, for which the operation time is long, and the operation steps are cumbersome, qPCR has had limited application in the field [21]. Loop-mediated isothermal amplification (LAMP) detection of ASFV is fast [22,23] and has been used extensively to diagnose epidemic diseases [24]. Isothermal amplification based on recombinase, such as recombinase polymerase amplification (RPA) developed by TwistDx (Cambridge, UK) [25] or recombinase-aided amplification (RAA) developed by Qitian (Wuxi, China) [26], have improved nucleic acid-based tests, providing fast, specific, diverse readouts without the use of thermal cyclers [27]. Real-time fluorescent RPA can quickly detect ASFV [28]. Verified by clinical samples, the qPCR assays incorporated into by RPA, RAA, and OIE have good diagnostic consistency, with the kappa values of 0.960 and 0.973 for RPA and RAA compared with OIE, respectively [27]. However, in contrast, the basic qPCR and LAMP assays are more widely used, and their reagents are easier to obtain, thus reducing the cost [29], representing the “gold standard” to detect the majority of viruses. Their sensitivity and specificity are higher than those of RPA and RRA assays, making them more suited to assess clinical samples with a low concentration of the target. Herein, we aimed to develop DNA extraction-free qPCR, visual LAMP, and fluorescent LAMP assays, which would be suitable to detect clinical samples in pig farms. We further aimed to verify the clinical performance of DNA extraction-free qPCR and LAMP assays and to compare them with OIE qPCR to detect field samples.

## 2. Materials and Methods

### 2.1. ASFV Reference Material and Clinical Samples

ASFV plasmid reference material (5.8 × 10^3^ copies/μL, GBW (E) 091034) was obtained from Beijing Tianzhitai Biotechnology Co., Ltd. (Beijing, China). Porcine parvovirus (PPV), porcine circovirus (PCV1), porcine reproductive and respiratory syndrome virus (PRRSV), and classical swine fever virus (CFSV) inactivated genomic DNA/RNA from clinical inactivated sample were obtained from Beijing Lambrui Biotechnology Co., Ltd. (Beijing, China). A total of 100 clinical samples, including EDTA-blood, spleen, lung, lymph node, kidney, tonsil, liver, and brain, were collected from domestic pigs in China. These samples were firstly detected by real-time PCR as recommended by the OIE at China’s animal disease prevention and control center (Beijing, China). Based on the result, in this study, a panel of 62 positive samples (including 12 weak positive cycle threshold (Ct) value, Ct ≥ 30) and 38 negative samples was adopted.

### 2.2. Heated Lysis (DNA Extraction-Free) of Clinical Samples

Clinical blood samples: To 5–20 μL of blood samples, 100 μL of mightyprep reagent (Code 9182, Takara Co., Ltd., Dalian, China) was added and mixed using an oscillator. The samples were then heated for 10 min at 95 °C before being centrifuged for 2 min at 12,000× *g*. The supernatant was retained and used directly as the template for qPCR and LAMP assays.

Clinical pig tissue samples: We took a 0.1–0.2 g sample of pig tissue, such as brain, kidney, liver, lymph node, and spleen; placed it in a grinding tube containing phosphate-buffered saline (PBS, pH 7.4); and ground it to make about 10% tissue homogenate. The homogenate was subjected to centrifugation for 5 min at 5000× *g*. A sample of the supernatant (5–20 μL) was added with 100 μL of mightyprep reagent, mixed, heated, and centrifuged as above. The supernatant was used directly as the template for qPCR and LAMP assays.

### 2.3. DNA Extraction

DNA was extracted from clinical samples for a DNA-extraction-based OIE qPCR assay. For clinical blood samples and clinical pig tissue samples, we took 5–20 μL of blood samples or 5–20 μL of supernatant of pig tissue samples that had been processed as above mentioned and extracted the DNA by using bead virus DNA/RNA extraction kit (DP438-T2k, Tiangen Biochemical Technology Co., Ltd., Beijing, China) according to the manufacturer’s instructions.

### 2.4. Oligonucleotides

Primers and probes for DNA extraction-free qPCR (Fw1/Rev1/Probe1 and Fw2/Rev2/Probe2) were used in this study (shown in Table 1). These primers and probes were designed for qPCR using Primer Premier software 6.0 (Applied Biosystems, Foster City, CA, USA), and the primer and probe combinations could cover 24 genotypes of the ASFV 72 gene. The primers for LAMP (F3/B3/FIP/BIP) and the loop primer (LB) used in this study were the same as those used in our previous article [30]. The primers and probe for OIE qPCR were those recommended by the OIE [31]. Takara Co., Ltd. synthesized the primers and probes.

### 2.5. OIE qPCR, DNA Extraction-Free qPCR, and DNA Extraction-Free LAMP Assay

The CFX96 real-time PCR system (BIO-DL, Auburn, AL, USA) was used to carry out the qPCR assay. A visual LAMP assay was performed on a MyBL-100 C dry heat thermostat (Yasuwang trading Co., Ltd., Shanghai, China), and fluorescent LAMP assay (with SYTO-9 fluorescent dye) was performed on the CFX96 real-time PCR system (BIO-DL, USA). This study summarized the reactions system and the thermal cycling program for OIE qPCR, DNA extraction-free qPCR, and DNA extraction-free LAMP assays in Table 2. In particular, DNA extraction-free qPCR accelerated the thermal lysis of the template through 15 cycles of pre-amplification and pre-amplification (15 cycles of 10 s at 95 °C and 10 s at 50 °C) during which we did not collect the FAM fluorescence signal.

### 2.6. Sensitivity Assay

The limit of detection (LOD) was determined for the DNA extraction-free qPCR and visual LAMP, fluorescent LAMP assays, and OIE qPCR assay. Serial dilutions of nucleic acid reference material with ASFV plasmid DNA were prepared at 5.8 × 10^3^, 5.8 × 10^2^, 5.8 × 10^1^, and 5.8 copies/μL. Four series of concentrations were measured. At least two or three replicates of each concentration were assayed.

### 2.7. Specificity Assay

The specificity for ASFV of the DNA extraction-free qPCR and visual LAMP, fluorescent LAMP assays, and OIE qPCR assay were evaluated using ASFV nucleic acid reference material and other viruses for DNA/RNA with similar symptoms (CFSV, PRRSV, PCV, PPV, and PRV). Two replicates for each sample were tested. Healthy pig tissues were used as negative controls, and H_2_O served as the blank control.

### 2.8. Comparison of DNA Extraction-Free qPCR and LAMP with OIE qPCR Using Clinical Samples

The veterinary service collected 100 samples during outbreaks of ASFV in 2018 and 2019. Using these samples, we compared the performances of DNA extraction-free qPCR, visual LAMP, and fluorescent LAMP assays with that of the DNA-extraction-based OIE qPCR. Two replicates for each sample were tested. The kappa value, determined using MedCalc software (MedCalc Software bvba, Ostend, Belgium), was used to measure the degree of agreement between the test results of these assays.

### 2.9. Statistical Analyses

Data are shown as the mean ± standard deviation. The analytical sensitivity of the assays to determine ASFV was assessed using semi-log regression analysis in GraphPad PRISM (GraphPad Inc., La Jolla, CA, USA). Probit regression analysis of the assay results was carried out using MedCalc at a 95% probability level (confidence interval, CI).

## 3. Results

### 3.1. Analysis of Sensitivity

**Figure 1:**
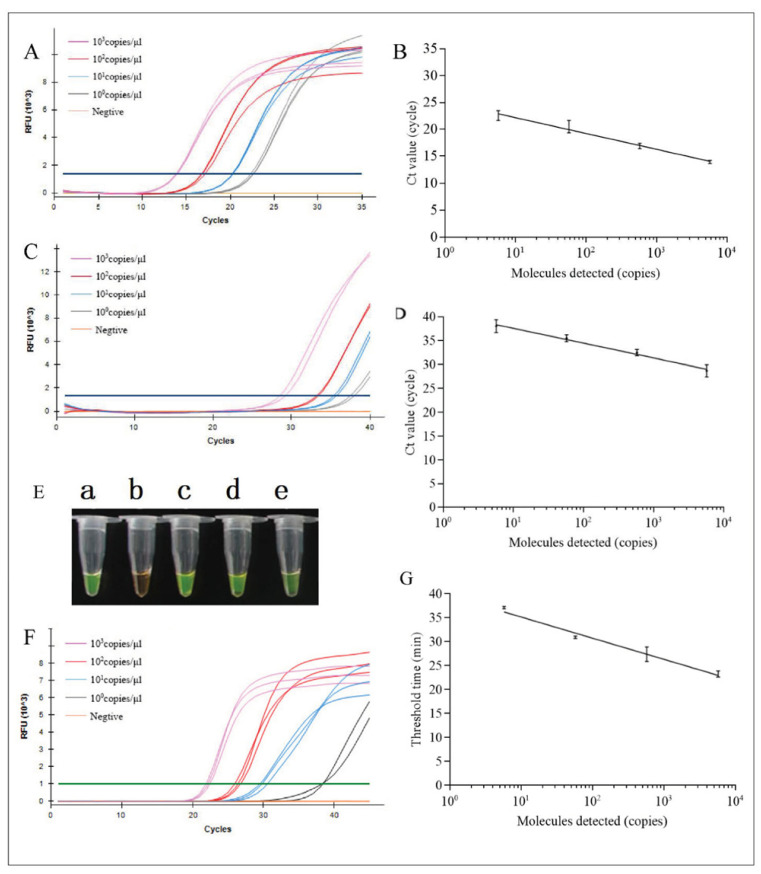
Detection sensitivity of African swine fever virus (ASFV) analyzed using DNA extraction-free qPCR, visual LAMP, and fluorescent LAMP, and OIE qPCR. Amplification plot (**A**) and linear correlation curve (**B**) of DNA extraction-free qPCR; amplification plot (**C**) and linear correlation curve (**D**) of OIE qPCR; sensitivity of visual LAMP (**E**) (a, 5.8 × 10^3^ copies/μL; b, negative; c, 5.8 × 10^2^ copies/μL; d, 5.8 × 10^1^ copies/μL; e, 5.8 copies/μL); amplification plot (**F**) and linear correlation curve of fluorescent LAMP (**G**). qPCR, quantitative real-time PCR; LAMP, loop-mediated isothermal amplification; OIE, world organization for animal health.

The sensitivities of DNA extraction-free qPCR, visual LAMP, and fluorescent LAMP assays and the OIE qPCR assay were assessed using serial dilutions of ASFV nucleic acid reference material of plasmid DNA. All four assays efficiently detected low ASFV levels (Table 1). The LOD of the DNA extraction-free qPCR assay (Figure 1A,B) was 5.8 copies/μL, and the OIE qPCR assay had a LOD of 5.8 copies/μL (Figure 1C,D). The LODs of the visual LAMP and fluorescent LAMP assays were both 5.8 copies/μL (Figure 1E–G). Compared with OIE qPCR (Ct value, 37.72 ± 0.39), the positive fluorescence signal of the DNA extraction-free qPCR assay was stronger (Ct value, 22.09 ± 0.14) for the LOD concentration of 5.8 copies/μL. However, the positive turbidity of visual LAMP was weak, which can easily lead to false-negative results. Only two of the three replicates of fluorescent LAMP were positive. Thus, DNA extraction-free qPCR could detect low copy more clearly, making it suitable to detect low virus concentrations.

### 3.2. Specificity Analysis

**Figure 2:**
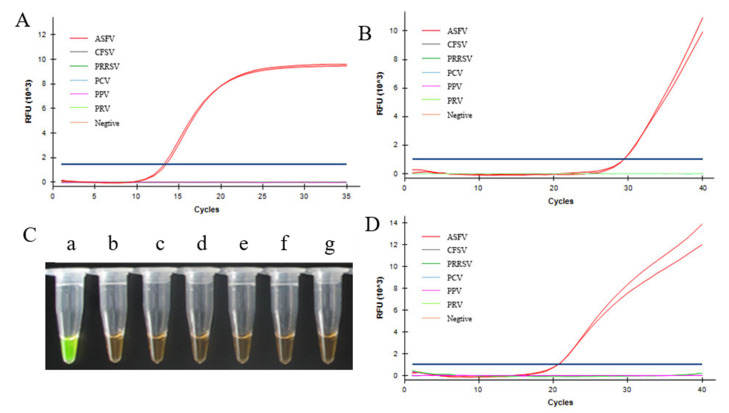
Specificity of ASFV detection by DNA extraction-free qPCR, visual LAMP and fluorescent LAMP, and OIE qPCR. Specificity results for DNA extraction-free qPCR (**A**), OIE qPCR (**B**), visual LAMP (**C**), and fluorescent LAMP (**D**) in detecting ASFV (**a**), CFSV (**b**), PRRSV (**c**), PCV (**d**), PPV (**e**), and PRV (**f**) and healthy pig tissues (**g**). CSFV, classical swine fever virus; PRRSV, porcine reproductive and respiratory syndrome virus; PCV, porcine circovirus; PPV, porcine parvovirus; PRV, porcine pseudorabies virus.

We tested the specificity for ASFV of DNA extraction-free qPCR, visual LAMP, and fluorescent LAMP assays in comparison with the OIE qPCR assay. ASFV nucleic acid reference material, along with CFSV, PRRSV, PCV, PPV, PRV, and healthy pig tissues, were used in the specificity test (Figure 2). All four assays only amplified ASFV, with no cross-reaction with any of the other viruses.

### 3.3. Performance of DNA Extraction-Free qPCR and LAMP for Clinical Samples Compared with OIE qPCR Testing

**Figure 3:**
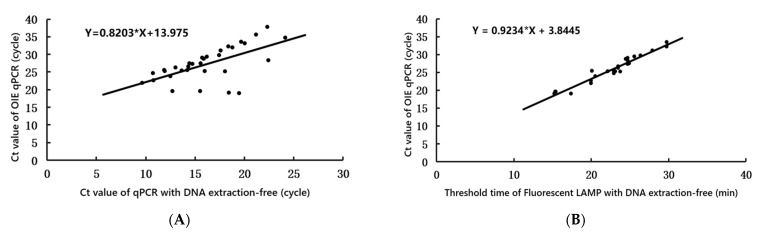
Comparison of clinical performance between the cycle threshold (Ct) value of ASFV for DNA extraction-free qPCR (*x*-axis) (**A**), the threshold time of fluorescent DNA extraction-free LAMP (*x*-axis) (**B**), and Ct value of OIE qPCR (*y*-axis) on positive clinical samples (*n* = 64).

To evaluate the practical application of DNA extraction-free qPCR, visual LAMP, and fluorescent LAMP assays to detect ASFV, 100 porcine samples of blood and tissue (brain, kidney, liver, lymph node, spleen) suspected to be positive for ASFV were assessed, and the results were compared with those obtained using OIE qPCR (Supplementary Table S1). Overall, using OIE qPCR, 64 samples were confirmed to be positive for ASFV DNA, with Ct values ranging from 18.32 to 37.77. Thirty-eight samples were confirmed to be negative, with undetermined Ct values. DNA extraction-free qPCR detected 64 samples as ASFV-DNA-positive (Ct values, 9.68–26.03) and 38 as negative (Ct value undetermined). DNA extraction-free visual LAMP indicated 54 positive and 46 negative samples, and DNA extraction-free fluorescent LAMP indicated 56 positive (threshold time, 13.22–28.74) and 44 negative samples (threshold time, undetermined). Analysis using linear correlation (Figure 3) showed that during detection, as the Ct value of OIE qPCR increased, the Ct values of DNA extraction-free qPCR (Figure 3A) and the threshold time of DNA extraction-free fluorescent LAMP (Figure 3B) increased correspondingly. Agreement analysis according to clinical sample detection (Table 1) demonstrated that the kappa value between DNA extraction-free qPCR and OIE qPCR was 1.0 (1~1, 95% CI), while between DNA extraction-free visual LAMP and DNA extraction-free fluorescent LAMP and OIE qPCR, the kappa values were 0.84 (0.73–0.94, 95% CI) and 0.88 (0.78–0.97, 95% CI), respectively. Additionally, in comparison to OIE qPCR (Table 3), the sensitivity and specificity of DNA extraction-free qPCR to identify ASFV was 100% (94.2–100%, 95% CI) and 100% (90.7–100%, 95% CI), respectively. For DNA extraction-free visual LAMP, the values were 87.1% (76.1–94.3%, 95% CI) and 100% (90.7–100%, 95% CI), respectively, and for DNA extraction-free fluorescent LAMP, the values were 90.32% (80.1–96.4%, 95% CI) and 100% (90.7–100%, 95% CI), respectively. Thus, DNA extraction-free qPCR and LAMP showed excellent diagnostic agreement with OIE qPCR to detect ASFV in clinical samples.

## 4. Discussion

The global pig industry (including that in China) is seriously threatened by ASFV [32]. A simple and sensitive diagnostic technique for early ASFV diagnosis in pig farms is required to prevent major losses to the pig industry [33]. qPCR is the “gold standard” for detecting most viruses. Due to its high sensitivity and specificity, together with the possibility for a high-throughput application, the PCR is a recommended method for screening and confirmation of suspected cases. Three validated PCR procedures for OIE are described below [31,34], consisting of a sample preparation followed by the test procedure. These procedures serve as a general guideline and a starting point for the PCR protocol. Optimal reaction conditions (incubation times and temperatures, models and suppliers of equipment, concentrations of assay reagents such as the primers and dNTPs) may vary, so the described conditions should be evaluated first. In recent years, constant temperature-amplification technologies without complex instruments and equipment have developed rapidly and have been gradually introduced to detect ASFV. The complexity of the current nucleic acid isolation methods limits their use outside of the modern laboratory environment [35]. This process is cumbersome and expensive, making it difficult to popularize in China’s pig farms. In addition, the operators of the farms lacked experience in extracting DNA, which affected the accuracy of ASFV test results. There have been few reports of ASFV detection in clinical samples based on nucleic-acid-extraction-free methods. In addition, there is a lack of research on the validation and comparison of different test methods for clinical samples.

Herein, DNA extraction-free qPCR, visual LAMP, and fluorescent LAMP assays were developed to quickly diagnose ASFV in clinical samples (pig blood and pig tissues). Our data showed that the specificities of the DNA extraction-free qPCR, visual LAMP, and fluorescent LAMP assays were all 100%, and their sensitivities were 100%, 87.1%, and 90.32%, respectively, indicating that these assays based on nucleic-acid-extraction-free techniques had good applicability in clinical practice, similar to that of OIE qPCR. In particular, DNA extraction-free qPCR accelerated the thermal lysis of the template through 15 cycles of pre-amplification to fully release the nucleic acid and could detect low copy more clearly. It also ensured the detection sensitivity based on the nucleic-acid-extraction-free technique from clinical samples, making it more suited to detecting clinical samples with low virus titers. In addition, our data showed that based on the detection sensitivity of an ASFV plasmid, visual LAMP and fluorescent LAMP assays could detect 5.8 copies/μL, the same as OIE qPCR. However, in the clinical performance verification, the sensitivities of DNA extraction-free visual LAMP and fluorescent LAMP were 87.1% and 90.32%, respectively, which were lower than those of DNA extraction-free qPCR and OIE qPCR. There was difference in matrix interference inhibition between plasmids and clinical samples, which indicated that LAMP assays have a weak anti-inhibition ability, and qPCR was more suitable for nucleic-acid-extraction-free assays and the detection of ASFV in clinical samples. The speed and simplicity of the methods make them ideally suited for molecular applications both within and outside the laboratory, including limited-resource settings.

This was the first comparative analysis of DNA extraction-free qPCR, visual LAMP, and fluorescent LAMP assays for clinical performance verification and detection of ASFV. Our validation results showed that these three methods were in good agreement with those of OIE qPCR (with kappa values of 1.0, 0.84, and 0.88, respectively). In particular, visual LAMP could be carried out using a simple thermostatic heater or thermostatic water bath facility, and detection could be completed within 30 min. Visual LAMP is the most simple of the three methods, but it is also the one with the lowest sensitivity. This eliminates the cumbersome operation steps of DNA extraction and is more suitable for ASF-run screening tests by inexperienced farmers in low-resource environments and, in the case of ASFV genome detection, can allow submission of samples for confirmation to accredited laboratory for accurate disease diagnostics, which is conducive to the rapid on-site detection of ASFV, making it a good choice for pig farms.

## Figures and Tables

**Table 1:** Primer and probe sequences of qPCR and LAMP primers for ASFV.

Assay	Primer	Sequence (5′–3′)	Reference
DNA extraction-free qPCR	Fw1	ATCCGATCACATTACCTA	This work
	Rev1	AGTGGAAGGGTATGTAAG	
	Probe1	(FAM)CCGTAACTGCTCATGGTATCAATCT(BHQ1)	
	Fw2	GCGATGATGATTACCTTTG	
	Rev2	CCCARCTAATATAAAAYTCTCTTG ^a^	
	Probe2	(FAM)ARCCACGGGAGGAATACCAAC(BHQ1)	
LAMP	F3	CGCAATATACGCTTTAAACCA	[30]
	B3	ACATTAGTTTTTCATCGTGGTG	
	FIP	AGGGGTTACAAACAGGTTATTGATGGGAGTCATTAATGAAATCTCGC	
	BIP	TACACAACCTTTTTGTAAAACGCGTATTGTTGGTGTGGGTCAC	
	LB	TCGCTTTTCGCTGATACGTG	
OIE qPCR	Positive Primer	CTGCTCATGGTATCAATCTTATCGA	[31]
	Negative Primer	GATACCACAAGATC(AG)GCCGT	
	Probe	(FAM)CCACGGGAGGAATACCAACCCAGTG(BHQ1)	

Abbreviations: ASFV, African swine fever virus. ^a^ Degeneracies: R (A + G); Y (A + T).

**Table 2:** The reactions system and the thermal cycling program for OIE qPCR, DNA extraction-free qPCR, and DNA extraction-free LAMP assays.

Assay	The Reactions (25 µL)	The Thermal Cycling Program	Reference
OIE qPCR	16 μL of qPCR mix containing enzyme (Code391A, Takara Co., Ltd.), 1 μL (0.4 μM) of positive primer, 1 μL (0.4 μM) of negative primer, 1 μL (0.4 μM) of probe, 4 μL of ultrapure water without DNase, and 2 µL of extracted DNA.	30 s at 95 °C, then 40 cycles of 5 s at 95 °C and 30 s at 60 °C; the fluorescent signals from FAM were collected at 60 °C.	[31]
DNA extraction-free qPCR	16 μL of qPCR mix containing enzyme (Code391A, Takara Co., Ltd., Dalian, China), 1 μL (0.4 μM) of forward primers (Fw1 and Fw2), 1 μL (0.4 μM) of reverse primers (Rev1 and Rev2), 1 μL (0.4 μM) of probe (Probe1 and Probe2), 4 μL ultrapure water without DNase, and 2 µL DNA extraction-free supernatant.	Pre-amplification (15 cycles of 10 s at 95 °C and 10 s at 50 °C) did not collect the FAM fluorescence signal; then, 1 min at 95 °C; followed by of 10 s at 95 °C and 30 s at 55 °C for 35 cycles; the fluorescent signals from FAM were collected at 55 °C.	This work
DNA extraction-free visual LAMP	12.5 μL of 2× reaction buffer, 1 μL of enzyme solution (Code 94001, Rongyan Biotechnology Co., Ltd., Beijing, China), 1 μL of visual MnCl_2_-calcein stock solution (Code SLP221, Rongyan Biotechnology Co., Ltd.), 1 μL (8 μM) of outer primer F3, 1 μL (8 μM) of outer primer B3, 1 μL (35 μM) of inner primer FIP, 1 μL (35 μM) of inner primer BIP, 1μL (15 μM) of loop primer LB, 0.5 μL of ultrapure water without DNase, and 5 μL of DNA extraction-free supernatant.	Initially, 63 °C for 30 min, followed by 95 °C for 2 min for termination. Under UV light (350–370 nm), samples that showed turbid green fluorescence were considered positive for ASFV, whereas samples with no turbidity were considered negative.	[30]
DNA extraction-free fluorescent LAMP	12.5 μL of 2× reaction buffer, 1 μL of enzyme solution (Code 94001, Rongyan Biotechnology Co., Ltd., Beijing, China), 1 μL of SYTO-9 fluorescent dye (No. 051011M, DHelixCo., Ltd., Guangzhou, China), 1 μL (8 μM) of outer primer F3, 1 μL (8 μM) of outer primer B3, 1 μL (35 μM) of inner primer FIP, 1 μL (35 μM) of inner primer BIP, 1 μL (15 μM) of loop primer LB, 0.5 μL of ultrapure water without DNase, and 5 μL of DNA extraction-free supernatant.	63 °C for 15 s, followed by 45 cycles at 63 °C for 45 s.	[30]

**Table 3:** Diagnostic performance comparison between qPCR, visual LAMP, DNA extraction-free fluorescent LAMP, and OIE qPCR.

Assays	Result	OIE qPCR with DNA Extraction			Performance Characteristics (%)		Agreement Kappa Value
		Positive	Negative	Total	Sensitivity	Specificity	
DNA extraction-free qPCR	Positive	62	0	62	100% (94.2–100%, 95% CI)	100% (90.7–100%, 95% CI)	1.0 (1–1, 95% CI)
	Negative	0	38	38			
	Total	62	38	100			
DNA extraction-free Visual LAMP	Positive	54	8	62	87.1% (76.1–94.3%, 95% CI)	100% (90.7–100%, 95% CI)	0.84 (0.73–0.94, 95% CI)
	Negative	0	38	38			
	Total	54	46	100			
DNA extraction-free Fluorescent LAMP	Positive	56	6	621	90.32% (80.1–96.4%, 95% CI)	100% (90.7–100%, 95% CI)	0.88 (0.78–0.97, 95% CI)
	Negative	0	38	38			
	Total	56	44	100			

## Data Availability

All datasets generated for this study are included in the article/supplementary material.

## Supplementary Materials

The following supporting information can be downloaded at: https://www.mdpi.com/article/10.3390/life12071067/s1, Table S1: Detection of ASFV by qPCR, visual LAMP, and fluorescent LAMP with DNA extraction-free in clinical samples.
