# Supplementary material for: Clinical Validation of DNA Extraction-Free qPCR, Visual LAMP, and Fluorescent LAMP Assays for the Rapid Detection of African Swine Fever Virus
Source: Life (Basel). 2022 Jul 16;12(7):1067. doi: 10.3390/life12071067 (PMC9320077; doi:10.3390/life12071067)
Supplement: Supplementary file 1 [file life-12-01067-s001.zip › life-1774610-supplementary.pdf]

Supplementary Table S1. Detection of ASFV by qPCR, visual LAMP, and fluorescent LAMP with DNA extraction-free in clinical samples.

Sample ID	Sample type	OIE qPCR with DNA extraction		qPCR with DNA extraction-free		Visual LAMP with DNA extraction-free	Fluorescent LAMP with DNA extraction-free	
		Ct value	Result	Ct value	Result	Result	Threshold time	Result
P1	Blood	28.77	+	15.91	+	+	24.45	+
P2	Blood	35.61	+	21.17	+	-	Undet.*	-
P3	Blood	28.98	+	15.75	+	+	24.73	+
P4	Blood	19.50	+	15.55	+	+	15.35	+
P5	Blood	18.94	+	19.48	+	+	15.24	+
P6	Blood	25.25	+	16.01	+	+	22.15	+
P7	Blood	31.95	+	18.8	+	-	Undet.	-
P8	Blood	28.29	+	22.42	+	+	24.7	+
P9	Blood	31.00	+	17.59	+	+	27.93	+
P10	Blood	25.28	+	18.03	+	+	23.74	+
P11	Blood	25.41	+	13.64	+	+	22.96	+
P12	Blood	37.77	+	22.32	+	-	Undet.	-
P13	Blood	25.45	+	11.88	+	+	20.13	+
P14	Blood	19.07	+	18.42	+	+	17.37	+
P15	Blood	29.35	+	16.22	+	-	25.55	+
P16	Blood	32.20	+	18.37	+	-	28.74	+
P17	Blood	19.51	+	12.74	+	+	15.41	+
P18	Blood	26.43	+	12.34	+	+	22.17	+
P19	Blood	19.64	+	16.41	+	+	17.41	+
P20	Blood	25.23	+	11.19	+	+	22.15	+
P21	Blood	19.32	+	16.41	+	+	14.12	+
P22	Blood	27.34	+	21.45	+	+	23.71	+
P23	Kidney	33.45	+	19.7	+	+	28.34	+
P24	Kidney	22.56	+	10.79	+	+	19.96	+
P25	Kidney	24.68	+	10.73	+	+	22.96	+
P26	Kidney	18.32	+	15.47	+	+	13.22	+

P27	Kidney	31.16	+	21.33	+	+	27.53	+
P28	Kidney	26.34	+	14.21	+	+	23.03	+
P29	Kidney	27.52	+	14.73	+	+	24.71	+
P30	Kidney	19.34	+	17.21	+	+	16.35	+
P31	Kidney	19.65	+	18.02	+	+	17.04	+
P32	Liver	26.58	+	14.32	+	+	23.47	+
P33	Liver	25.21	+	11.94	+	+	23.13	+
P34	Liver	33.15	+	20.03	+	-	Undet.	-
P35	Liver	27.56	+	14.98	+	+	24.19	+
P36	Liver	26.32	+	13.65	+	+	23.28	+
P37	Liver	33.67	+	19.72	+	+	28.25	+
P38	Liver	31.05	+	22.45	+	+	25.49	+
P39	Liver	19.32	+	17.41	+	+	16.33	+
P40	Liver	23.1	+	12.91	+	+	20.09	+
P41	Liver	21.37	+	10.43	+	+	18.16	+
P42	Liver	21.83	+	11.42	+	+	18.34	+
P43	Liver	35.21	+	26.03	+	-	Undet.	-
P44	Liver	24.34	+	11.57	+	+	21.91	+
P45	Liver	23.56	+	12.62	+	+	20.49	+
P46	Lymph node	26.19	+	13.03	+	+	23.47	+
P47	Lymph node	34.63	+	24.18	+	-	Undet.	-
P48	Lymph node	25.56	+	14.21	+	+	22.96	+
P49	Lymph node	27.03	+	13.75	+	+	22.49	+
P50	Lymph node	19.34	+	16.32	+	+	15.34	+
P51	Lymph node	21.93	+	9.68	+	+	19.98	+
P52	Lymph node	27.39	+	14.46	+	+	24.70	+
P53	Spleen	29.70	+	17.45	+	+	26.35	+
P54	Spleen	26.75	+	15.32	+	+	24.06	+
P55	Spleen	26.38	+	14.41	+	+	23.17	+
P56	Spleen	25.21	+	14.32	+	+	21.32	+
P57	Spleen	29.87	+	17.49	+	+	25.31	+
P58	Spleen	23.53	+	12.62	+	+	20.38	+
P59	Spleen	26.43	+	14.37	+	+	23.43	+

P60	Spleen	27.40	+	15.56	+	+	24.91	+
P61	Spleen	27.31	+	14.77	+	+	24.71	+
P62	Spleen	23.81	+	12.53	+	+	20.56	+
N1	Blood	Undet.	-	Undet.	-	-	Undet.	-
N2	Blood	Undet.	-	Undet.	-	-	Undet.	-
N3	Blood	Undet.	-	Undet.	-	-	Undet.	-
N4	Blood	Undet.	-	Undet.	-	-	Undet.	-
N5	Blood	Undet.	-	Undet.	-	-	Undet.	-
N6	Blood	Undet.	-	Undet.	-	-	Undet.	-
N7	Blood	Undet.	-	Undet.	-	-	Undet.	-
N8	Blood	Undet.	-	Undet.	-	-	Undet.	-
N9	Blood	Undet.	-	Undet.	-	-	Undet.	-
N10	Blood	Undet.	-	Undet.	-	-	Undet.	-
N11	Blood	Undet.	-	Undet.	-	-	Undet.	-
N12	Blood	Undet.	-	Undet.	-	-	Undet.	-
N13	Blood	Undet.	-	Undet.	-	-	Undet.	-
N14	Blood	Undet.	-	Undet.	-	-	Undet.	-
N15	Blood	Undet.	-	Undet.	-	-	Undet.	-
N16	Blood	Undet.	-	Undet.	-	-	Undet.	-
N17	Blood	Undet.	-	Undet.	-	-	Undet.	-
N81	Blood	Undet.	-	Undet.	-	-	Undet.	-
N19	Blood	Undet.	-	Undet.	-	-	Undet.	-
N20	Blood	Undet.	-	Undet.	-	-	Undet.	-
N21	Blood	Undet.	-	Undet.	-	-	Undet.	-
N22	Brain	Undet.	-	Undet.	-	-	Undet.	-
N23	Brain	Undet.	-	Undet.	-	-	Undet.	-
N24	Brain	Undet.	-	Undet.	-	-	Undet.	-
N25	Kidney	Undet.	-	Undet.	-	-	Undet.	-
N26	Kidney	Undet.	-	Undet.	-	-	Undet.	-
N27	Kidney	Undet.	-	Undet.	-	-	Undet.	-
N28	Liver	Undet.	-	Undet.	-	-	Undet.	-
N29	Liver	Undet.	-	Undet.	-	-	Undet.	-
N30	Liver	Undet.	-	Undet.	-	-	Undet.	-

N31	Lymph node	Undet.	-	Undet.	-	-	Undet.	-
N32	Lymph node	Undet.	-	Undet.	-	-	Undet.	-
N33	Lymph node	Undet.	-	Undet.	-	-	Undet.	-
N34	Lymph node	Undet.	-	Undet.	-	-	Undet.	-
N35	Spleen	Undet.	-	Undet.	-	-	Undet.	-
N36	Spleen	Undet.	-	Undet.	-	-	Undet.	-
N37	Spleen	Undet.	-	Undet.	-	-	Undet.	-
N38	Spleen	Undet.	-	Undet.	-	-	Undet.	-

* Undet., no amplification detected.
